# Postpartum hemorrhage in electronic health records: risk factors at admission and in-hospital occurrence

**DOI:** 10.61622/rbgo/2024AO14

**Published:** 2024-03-15

**Authors:** Raíssa Isabelle Leão Martins, Jussara de Souza Mayrink Novais, Zilma Silveira Nogueira Reis

**Affiliations:** 1 Universidade Federal de Minas Gerais Faculty of Medicine Department of Gynecology and Obstetrics Belo Horizonte MG Brazil Department of Gynecology and Obstetrics, Faculty of Medicine, Universidade Federal de Minas Gerais, Belo Horizonte, MG, Brazil.

**Keywords:** Postpartum hemorrhage, Puerperal disorders, Maternal mortality, Risk factors, Electronic health records

## Abstract

**Objective:**

Postpartum hemorrhage (PPH) is the leading cause of maternal death globally. Therefore, prevention strategies have been created. The study aimed to evaluate the occurrence of PPH and its risk factors after implementing a risk stratification at admission in a teaching hospital.

**Methods:**

A retrospective cohort involving a database of SISMATER® electronic medical record. Classification in low, medium, or high risk for PPH was performed through data filled out by the obstetrician-assistant. PPH frequency was calculated, compared among these groups and associated with the risk factors.

**Results:**

The prevalence of PPH was 6.8%, 131 among 1,936 women. Sixty-eight (51.9%) of them occurred in the high-risk group, 30 (22.9%) in the medium-risk and 33 (25.2%) in the low-risk group. The adjusted-odds ratio (OR) for PPH were analyzed using a confidence interval (95% CI) and was significantly higher in who presented multiple pregnancy (OR 2.88, 95% CI 1.28 to 6.49), active bleeding on admission (OR 6.12, 95% CI 1.20 to 4.65), non-cephalic presentation (OR 2.36, 95% CI 1.20 to 4.65), retained placenta (OR 9.39, 95% CI 2.90 to 30.46) and placental abruption (OR 6.95, 95% CI 2.06 to 23.48). Vaginal delivery figured out as a protective factor (OR 0.58, 95% CI 0.34 to 0.98).

**Conclusion:**

Prediction of PPH is still a challenge since its unpredictable factor arrangements. The fact that the analysis did not demonstrate a relationship between risk category and frequency of PPH could be attributable to the efficacy of the strategy: Women classified as "high-risk" received adequate medical care, consequently.

## Introduction

Postpartum hemorrhage (PPH) is a birth complication present in approximately 2 to 10% of all deliveries.^(1)^ The main cause of PPH is uterine atony, which is defined by the inability of the uterus to contract properly to control bleeding after delivery. Prolonged labor, hypertensive disorders, uterine overdistension, chorioamnionitis and anomalous placentation are some of the etiologies most associated with uterine atony.^(2)^ Annually, the estimated occurrence is 14 million cases of PPH with 140,000 related deaths, which represents one death every four minutes.^(3)^ In addition to the health impact, maternal morbidity and mortality related to PPH also has a meaningful social impact since most are young women who play a central role in their families.

It is already known that maternal death from PPH is preventable in most cases.^(4)^ Several strategies and management protocols have been established for PPH prevention, directing rapid risk identification and more suitable treatment of PPH. Among the prevention measures are the risk stratification of the pregnant woman for PPH at hospital admission, the recommendation for oxytocin administration after cord clamping, and active management of the placenta delivery.^(5)^ It is critical that gaps in obstetric care are identified and corrected. Strategies to combat PPH must be appropriate for local realities, including special attention to the population residing in remote areas with little access to health technologies. Especially in developing countries, women are particularly at risk for dying of a PPH.^(6,7)^ In Africa and Asia, the percentage of mortality from PPH reaches 30.8% and 33.9% respectively.^(8)^

Zero Maternal Deaths by Hemorrhage (0MMxH) is a project of the Pan American Health Organization/World Health Organization (PAHO/WHO) dedicated to the prevention of obstetric hemorrhage. The goal is to improve the quality of healthcare: eliminating barriers to access to health care services, qualifying health care professionals to deal with obstetric hemorrhage, and ensuring the availability of the medical supplies and equipment needed to deal with severe forms of PPH.^(4)^ Facing the challenges to mitigate PPH occurrence, this study aims to analyze the frequency of PPH in a reference maternity hospital. We evaluated the frequency of women with the low, medium and high risk for PPH by the 0MMxH classification.^(9)^ We also compared pregnant women who presented PPH with those who did not present to establish predictive variables of this in-hospital complication.

## Methods

### Study Design, Setting and Population

This is a retrospective cohort analysis using an electronic clinical database study involving all deliveries that took place at the Hospital das Clínicas of the Federal University of Minas Gerais (UFMG) from September 2019 to December 2020. Under ethical approval number CAAE55087421.0.0000.5149, this study accessed an unnamed database. Data collection occurred using structured clinical data which is routinely collected during pregnant women's in-hospital stay using an electronic medical records system, Sismater®.^(10)^ The framework of clinical variables retrieved for this study is available in supplementary table S1. This cohort analysis included all deliveries that occurred during the study period that met eligibility criteria. The inclusion criteria were gestational age at birth over 22 weeks or birth weight greater than or equal to 500 grams in the absence of such information: living or dead fetus. In addition, exclusion criteria were critical missing data that prevented classifying childbirth or abortion, such as the mode of delivery and birth weight. In the case of multiple gestations, we consider the mother once and only the weight of the first twin. The period of analysis was concurrent with the introduction of the 0MMxH in the maternity ward, which included an interface in the system dedicated to the registration of risk factors, with immediate classification into absent or low risk, medium risk, and high-risk PPH (Chart 1). According to each risk category, the staff became aware of the necessary systematization of preventable approaches to avoid or mitigate PPH. The medical staff was directly responsible for conducting the delivery in the maternity hospital. This university service works under clinical protocol recommendations based on best practices for delivery care.

**Chart 1:** Grades of PPH risk according to the introduced prevention program (at least one factor)

Low-risk	Medium-risk	High-risk
Absent of uterine scar	Previous C-section or uterine scar	Placenta previa
Single pregnancy	Mild preeclampsia	Severe preeclampsia
Equal to or less than three vaginal births	Uterine overdistension^a^	Hematocrit below 30%
Absent clotting disorder	Equal to or more than four vaginal births	Platelets below 100,000/mm^3^
No previous PPH	Chorioamnionitis	Active vaginal bleeding at admission
	Previous uterine atony or any obstetric hemorrhage	Coagulopathy
	Obesity (BMI> 35kg/m^2^)	Using anticoagulant
		Placental abruption
		Placenta accreta
		Two or more medium risk factors

a Multiple gestations, polyhydramnios, fetal macrosomia; PPH - postpartum hemorrhage; BMI - body mass index

PPH was identified as a visual estimation of blood loss greater than 500 mL from the genital tract in the first 24 hours after vaginal delivery or greater than 1000 mL after cesarean delivery.^(11)^ Previous uterine scar joined the previous C-section or a history of uterine intervention. Placenta accreta refers to antenatal ultrasonography diagnosis.

### Statistical analysis

Categorical variables were described in terms of frequency and the numerical variables in the form of measures of central position (mean, median) and variability (standard deviation). The comparisons analysis between groups used the Mean-t Test, Mann-Whitney Test, Chi-square, and Fisher Test T, according to variables and frequency of distribution. The variables chosen for comparison took into account those present in the risk classification of the 0MMxH. Multivariate modeling with logistic regression was employed to estimate the raw odds ratio (OR) of PPH adjusted for cofactors, accompanied by 95 % confidence intervals (CI). For this step, we included all predictor variables from the univariate analysis, considering input a p-value of 0.10. All sets of variables had been adjusted OR using a simple enter method. The fit of the models and calibration, specifically the Hosmer-Lemeshow goodness-of-fit test, and the coefficient of determination (adjusted R^2^) were carried out based on the hypothesis that all coefficients were 0. The statistical program SPSS® 22.0 was used for the analysis.^(12)^ The significance level adjusted for the hypothesis test was 5 %.

## Results

**Figure 1:**
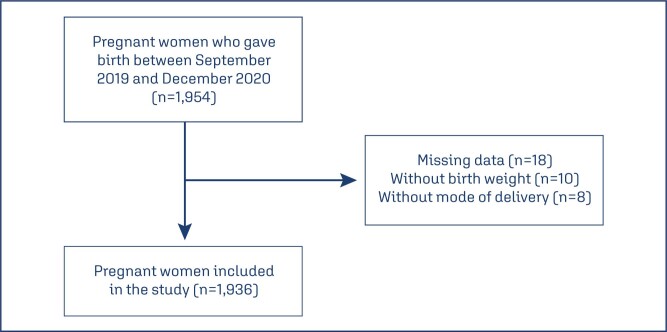
Flowchart of patient allocation according to eligibility criteria

During the period between September 2019 and December 2020, we assessed 1954 registers of pregnant women who gave birth (Figure 1). Eighteen women (0.92%) were excluded due to missing data. Therefore, 1936 women who gave birth to 1953 newborns were included in the analysis. Among these, 17 (0.88%) gave birth on the way until maternity and were admitted during early-postpartum. Concerning maternal complications during hospital stay, 28 (1.4%) women had transfusional intervention due to proper indications, 2 (0.1%) uterine rupture, and one maternal death (0.5 / 1000 women). Thirty-three (1.7%) of concepts were stillbirths for any reason; first-minute Apgar had a median of 8.0 (IQR: 1), and five-minute Apgar had a median of 9.0 (IQR: 0).

The prevalence of PPH was 6.8% (131 participants). Among patients considered at high risk for PPH, 8.19% actually had PPH with no significant difference in frequency compared to medium risk (5.36%) or low risk (6.6%). This distribution was not linked to the classification risk category (p=0.119). In table 1 demographic characteristics of participants are shown, comparing them between two groups, according to the occurrence of PPH. The mean age was 29.7 years with a standard deviation of 7 years. Regarding parity, 40.3% were nulliparous, with no difference between both groups. Among the participants, 20.7% presented a previous C- section and 20.6% presented a previous uterine scarring, which did not differ between participants with and without PPH. As expected, the occurrence of multiple gestations was higher among women who presented PPH. History of diabetes; hypertensive disorders of pregnancy, thrombocytopenia and use of anticoagulants were not different between women with or without PPH.

**Table 1:** Demographic profile of participants with PPH and without PPH, at maternity admission

Variables	Total (n=1936) n(%)	PPH (n=131) n(%)	Without PPH (n=1805) n(%)	p-value
Age, years, mean (SD)	29.7(7.0)	30.4(7.1)	29.6(7.0)	0.792*
Nulliparous	724(40.3)	47(35.9)	677(40.6)	0.817**
Previous C-section				0.176**
0	1535(79.3)	112(85.5)	1423(78.8)	
1	275(14.2)	14(10.7)	261(14.5)	
2 or more	126(6.5)	5(3.8)	121(6.7)	
Previous uterine scar	404(20.6)	19(14.5)	385(21.6)	0.063**
Multiple pregnancy	57(3.3)	9(6.9)	48(3.0)	0.019**
Hypertensive disorders of pregnancy	384(19.8)	33(25.2)	351(19.4)	0.111**
Diabetes	298(16.7)	20(15.3)	278(16.8)	0.657**
Thrombocytopenia	16(0.9)	3(2.3)	13(0.8)	0.078**
Use of anticoagulant	29(1.6)	2(1.5)	27(1.6)	0.927**

SD: standard deviation.

PPH: postpartum hemorrhage.

* Mean-t Test.

** Qui-square Test

Table 2 shows information about delivery, obstetric interventions, and intrapartum complications, comparing participants with and without PPH. The rate of interventions was 17.2% for oxytocin during first and/or second stages of labor, and 21.4% for analgesia. Considering only 1,125 vaginal births, the frequency of forceps was 3.5% and vacuum-extractor was 3.9% - both interventions were individually associated with PPH occurrence. The median of gestational age at delivery was 37.6 weeks (interquartile of 2 weeks). The mean weight of newborns was 2,947 grams (standard deviation of 657 grams). Both data did not differ among participants with or without PPH. The occurrence of spontaneous or induced labor was also the same between groups. Participants with PPH were more likely to present active bleeding on admission and to be exposed to oxytocin and analgesia during labor. Placenta accreta, placental abruption and retained placenta were more prevalent in participants with PPH, as was the frequency of obstetric anal sphincter injuries (OASIS). The frequency of vaginal delivery and C-section was different between groups of PPH with a significant difference of more cases of PPH in those who had a vaginal delivery.

**Table 2:** Delivery, obstetric interventions and intrapartum complications, compared between participants with and without PPH

Variables	Total (n=1936) n(%)	PPH (n=131) n(%)	Without PPH (n=1805) n(%)	p-value
Gestational age, weeks, median (IQR)	37.6(2.0)	37.2(3.0)	38.0 (2.0)	0.137*
Birth weight, grams, median (IQR)^a^	2947(657)	2910(712)	2950 (653)	0.510*
Labor****				0.342**
Spontaneous	721(40.1)	45(34.3)	676(40.5)	
Induced	489(27.2)	41(31.3)	448(26.9)	
Without labor	589(32.7)	45(34.4)	544(32.6)	
Active bleeding on admission	11(0.6)	5(3.8)	6(0.4)	0.001**
Non-cephalic presentation	133(7.4)	15(11.5)	118(7.1)	0.067**
Oxytocin during first and/or second stages of labor	305(17.2)	35(26.7)	270(16.4)	0.003**
Analgesia during labor	382(21.4)	42(32.1)	340(20.6)	0.002**
Retained placenta^b^	15(0.8)	7(5.3)	8(0.4)	<0.001**
Placenta previa	11(0.6)	2(1.5)	9(0.5)	0.160**
Placenta accreta	5(0.3)	2(1.5)	3(0.2)	0.046***
Placental abruption	19(1.0)	7(5.3)	12(0.7)	<0.001**
Vaginal delivery*****	1125(58.7)	89(67.9)	1036(58.1)	0.027**
C-section	790(41.3)	42(32.1)	748(41.9)	
Forceps during vaginal birth	39(2.2)	7(5.3)	32(1.9)	0.010**
Vacuum-extractor	44(2.5)	8(9.0)	35(2.1)	0.001**
OASIS^c^	29(2.6)	3(3.4)	26(2.5)	0.624**

IQR - interquartile range; PPH - postpartum hemorrhage;

* Mann-Whitney Test;

** Qui-square Test;

*** Fisher Exact Test;

**** 137 missing data;

***** 21 missing data;

a In case of multiple gestation, only the first twin;

b The placenta is not delivered within 30 minutes of childbirth;

c Third and fourth-degree tear of perineum, considering only vaginal deliveries

A multivariate analysis in table 3 was done considering variables presenting p-value above 0.10: previous uterine scar, multiple pregnancy, thrombocytopenia, active bleeding on admission, fetal presentation (cephalic and non-cephalic), oxytocin during labor, analgesia during labor, C-section vs. vaginal delivery, instrumental delivery, retained placental, placenta accreta, placental abruption. According to this analysis, the adjusted OR for PPH was significantly higher in participants who presented multiple pregnancy (OR 2.84, 95%CI: 1.26 to 6.42), active bleeding on admission (OR 5.81, 95%CI: 1.29 to 26.17), non-cephalic presentation (OR 2.40, 95%CI: 1.23 to 4.74), retained placenta (OR 9.46, 95%CI: 2.90 to 30.81) and placental abruption (OR 6.85, 95%CI: 2.03 to 23.15). Vaginal delivery figured out as a protective factor, with an odds of 0.58 (0.34 to 0.98). Analgesia and oxytocin, when corrected by other factors, had no significant effect on PPH.

**Table 3:** Risk factors of PPH in a multivariate analysis

Risk factor	Crude OR (95%CI)	Adjusted OR (95%CI)
Previous uterine scar	0.63 (0.38 to 1.03)	0.84 (0.46 to 1.53)
Multiple pregnancy	2.35 (1.13 to 4.90)	2.84 (1.26 to 6.42)
Thrombocytopenia	2.97 (0.83 to 10.54)	3.05 (0.78 to 11.99)
Active bleeding on admission	10.9 (3.28 to 36.23)	5.81 (1.29 to 26.17)
Fetal presentation, non-cephalic	1.69 (0.96 to 2.99)	2.40 (1.23 to 4.74)
Oxytocin during first and/or second stages of labor	1.86 (1.24 to 2.80)	1.38 (0.85 to 2.25)
Analgesia during labor	1.82 (1.24 to 2.68)	1.29 (0.80 to 2.09)
Vaginal delivery	1.53 (1.05 to 2.24)	0.58 (0.34 to 0.98)
Instrumental delivery ^a^	3.03 (1.61 to 5.68)	2.14 (1.08 to 4.25)
Retained placenta	12.68 (4.52 to 35.54)	9.46 (2.90 to 30.81)
Placenta accreta at admission	8.54 (1.41 to 51.55)	2.53 (0.29 to 22.46)
Placental abruption	8.44 (3.26 to 21.81)	6.85 (2.03 to 23.15)

OR - odds ratio; CI - confidence interval; PPH - postpartum hemorrhage; Hosmer-Lemeshow p = 0.992. a forceps or vacuum extractor

## Discussion

The main contribution of our study was reinforcing the magnitude of the PPH (6.8%) in a sample of Brazilian pregnant women and rising risk factors at hospital admission as well as those presented during or after delivery. PPH is one of the commonest causes of preventable pregnancy-related death, causing 25% of maternal deaths worldwide.^(13-15)^ But the prevalence varies according to the country analyzed, considering that successive improvements in maternity care during the 20th century have led to an impressive decline in the overall maternal mortality in high-income countries. In low income countries, PPH is still a challenge to be overcome. Meanwhile, in Spain the frequency of PPH is 3%, and in Ethiopia it is 8.24%.^(8,16)^

In our teaching-maternity, most childbirths occurred vaginally (58.7%) and vaginal delivery increased 73% the chance of PPH when analyzed alone. However, adjusted to other predictors in a multivariate analysis, such effect was corrected for some protection, even though the upper confidence interval almost reached value 1. The outcome corroborated previous reports pointing to the risk of bleeding greater with C-section.^(6)^ Otherwise, an increased risk of PPH associated with vaginal delivery in low- and middle-income countries has previously been reported. In a multicountry study, C-section was associated with reduced PPH in non-African settings.^(17)^ Comparing our results with the synthesis of evidence, reported by Ende et al. (2021),^(18)^ uterine atony is the central core of the pathological process; instead, cesarean delivery remains unclear. Other points of attention for interpretation of our results deserve highlight. This teaching-maternity adopts bleeding greater than 500 mL after vaginal deliveries and greater than 1000 mL after C-sections as definitions for PPH in accordance with ACOG (American College of Obstetricians and Gynecologists). Therefore, since the diagnosis of PPH is based on a visual estimate, maybe the cases of PPH after C-sections are being underestimated by the obstetrician-assistants. The other point is the profile of pregnant women in the birth scenario of analysis, where women, even with vaginal deliveries, had more comorbidities than the general population of parturients.^(19)^

Among the analyzed intrinsic factors previous to the birth, maternal age, nulliparity, previous C-section or any uterine scar, and gestational age had no association with PPH in this cohort. In fact, previous studies reported that a substantial amount of PPH occurs in the absence of recognized risk factors.^(18)^ Besides, the epidemiology of in-hospital PPH ranges according to the birth scenario, access to obstetrical assistance, socioeconomic status, and the quality of facilities.^(20)^ In our study, pregnant women have assisted under evidence-based protocols for the route of delivery, considering the risks and maternal and fetal conditions associated with gestational complications. We attribute these results to good obstetric practices such as bathing for pain relief, active management of the placenta, postpartum oxytocin, compliance with the right to a companion, and follow-up of C-section rates supported by Robson 10 groups monitoring.^(21,22)^

Previous diseases as hypertensive disorders of pregnancy and diabetes had no association with PPH in this Brazilian sample. But transition countries such as Brazil have maternal and child health indicators with large internal variations, depending on the quality and supply of health services. We know that the expected evolution is that the direct causes of maternal death (including hemorrhage) are greatly reduced with investments in health, as observed in rich countries. We believe that the data analyzed in a quaternary referral service reflect this transition in the quality of care, in such a way that hypertension and diabetes better addressed were not associated with PPH. With regard to the analyzed intrinsic factors prior to birth, the presence of thrombocytopenia was higher among pregnant women who presented PPH. However, in the multivariable analysis there was not statistical significance. A possible reason for the lack of statistical significance in our result would be a small sample size with this finding. Rottenstreich et al.^(22)^ carried out a retrospective study with primiparous women that demonstrated a higher risk of PPH among women with mean platelet count between 100,000 and 150,000.^(23)^

Regarding labor variables, univariate analysis revealed a borderline significant association between non-cephalic presentations and PPH (p=0.067). Nevertheless, in the multivariate analysis, this presentation was consistently associated with PPH since the chance of PPH was 2.4 times higher compared to cephalic presentation. This finding is consistent with a multicountry study from 436,112 deliveries.^(24)^ In their cohort of deliveries, non-cephalic presentation was associated with a significantly increased risk of PPH, especially in vaginal delivery compared with C-section. Also in accordance with what was demonstrated in our study, it is already well established that conditions that over distend the uterus such as multiple gestations are associated with impaired uterine contractility and thus atonic hemorrhage. A population-based registry study of 307,415 women giving birth found that multiple pregnancies increase the chance of major bleeding by 2.34 times.^(25)^ Otherwise, birth weight was not associated with PPH in present analysis. However, further analysis with a larger sample deserves comparing ranges of weight to confirm the lack of association.

When it comes to birth assistance in general, there is a common sense that caregivers should make efforts to minimize the need for medical interventions during childbirth, reducing them to what is strictly necessary.^(26)^ Hence, operative vaginal delivery was associated with PPH. The frequency of operative vaginal delivery was 7.4%, and using forceps or vacuum-extractor raised 2.14 the chance of PPH. In other reports, instrumental delivery rates range from 1.5% to 15% in different countries.^(27,28)^ Therefore, the frequency of operative vaginal delivery in our birth scenario was not out of expectation. A possible explanation is that prolonged labor, which is an independent risk factor for PPH, would be associated with a higher proportion of operative vaginal delivery and, then, with PPH.^(29)^ We did not have access to the timeline of labor in our study to compare; however, this topic could be explored better in a further study. Besides, such instrumental assistance is an emergency care and part of complications relied on the motives for the operative intervention *per se*.

Use of oxytocin during labor and analgesia was associated with PPH in our univariate analysis, but not when adjusted to the other predictors. This finding is contradictory with most studies that demonstrated an increase in PPH rates with interventions with oxytocin use and analgesia. The rate of analgesia in the total sample was 21.4%, and oxytocin augmentation was 17.2%, numbers as expected since a systematic review showed that rates of oxytocin use can range from 0-60% and epidural use from 0-84%.^(30)^ A possible explanation for the absence of correlation in our study is variations on optimal time for the intervention and dose of drug administration. So, depending on the expertise of obstetricians and anesthesiologists, the use of oxytocin and analgesia can help or not the labor evolution and therefore predispose or not to PPH.

Unsurprisingly, active bleeding on admission was associated with PPH. The reason is easy to comprehend thinking about its possible etiologies as placental abnormalities or coagulation disorders.^(31,32)^ Placental abruption can result in a life-threatening hemorrhage. In present analysis this occurrence increased 6.85 times the chance of PPH, even adjusted to the other factors. For comparison, a meta-analysis showed that placental disorders including placenta previa or placental abruption increased the risk of PPH by 2.74-fold.^(18)^ In addition, also retained placenta was more prevalent in participants with PPH, considering that some women require manual placenta extraction. Manual extraction of placenta may lead to massive hemorrhage with hemodynamic instability requiring emergency interventions including blood transfusion, interventional radiology and hysterectomy.^(33)^ A recent prospective cohort study demonstrated high rates of PPH in vaginal delivery followed by pathological confirmation of retained placenta and our findings pointed to a chance of 9.46 higher PPH when the placenta delays more than 30 minutes to delivery.^(34)^ Otherwise, our study did not correlate previously known placenta accreta on admission with PPH. A possible interpretation is that the systematic preventive approach, including risk stratification were successful towards avoiding PPH.

As the study comprised a retrospective cohort, there are some limitations, mainly data loss. However, only eighteen registries were wasted. Furthermore, biases were minimized as data collection was performed by the physician in parallel with the provision of care, by filling out a structured electronic medical record. Even so, additional studies are necessary since prediction of PPH is still a challenge. The minority of women with some risk factors will develop PPH and, of PPH cases, a considerable part will occur in patients classified as low-risk. The risk assessment of the 0MMxH proposed in Brazil in 2017 by Osanan et al.^(3)^ is part of a core aspect of public health practice. Is an important step towards better outcomes and improvements of healthy indicators in the context of PPH. It is linked to anticipatory planning measures, like to transfer high-risk women to specialized centers, mobilization of experienced staff, resources, and blood products in anticipation of PPH. Risk assessment of PPH is also linked to secondary prevention, including close monitoring of high-risk patients after delivery.^(31)^ Future studies with specific subgroup analysis (modes of delivery, primiparous or multiparous woman) would improve our knowledge around our local center, but with the prospect of being able to assist other countries with a common goal of reducing maternal deaths from PPH. Another point was the lack of comparisons with 0MMxH risk classification pre-deployment data, the absence of a close relationship between risk category classification and frequency of PPH can be a result of better decisions made. Since our study is not a validation study of the 0MMxH, a PPH temporal series analysis could better analyze the evolution of PPH occurrence over time and the impact of preventive interventions. Among the main conclusions about the causes of the great improvement in mortality rates are the expansion of access to prenatal care, with early diagnosis and well-defined treatment flow for diagnosed cases.^(35)^

## Conclusion

When it comes to PPH, an acute and sometimes unpredictable event, the way to reduce its prevalence and the number of fatal victims certainly involves identifying the most exposed groups and training the healthy team, with well-defined flows, conducts homogeneous services and effective communication between services. Identifying possible flaws in this process is an instrument capable of motivating improvements that can mean saved lives. The main contribution of our study was to reinforce the magnitude of PPH in a sample of Brazilian pregnant women, the prevalence was 6.8% (131 participants). Multiple pregnancy, active bleeding on admission, non-cephalic presentation, retained placenta, placental abruption and C-section delivery were identified as risk factors for PPH.

## Table S1 Database of clinical variables collected from each childbirth

**Table:**

ID_HOSPITALIZATION	PATIENT IDENTIFICATION	INTEGER
AGE WOMEN	MOTHER'S AGE	INTEGER
PPH		YES / NO
PREGNANCY	INT 1 -SINGLE, 2 - TWINS, 3 - TRIPLETS, 4 - QUADRIGEMINS, 5 - QUINTUPLES	INTEGER
FETAL PRESENTATION	1 - CEPHALIC, 2 - BREECH, 3 - ANOMALOUS (TRANSVERSE OR OBLIQUE))	INTEGER
LABOR ON ADMISSION	1- SPONTANEOUS, 2- INDUCED, 3- OUT OF LABOR	INTEGER
PARITY	1 - NULIPAROUS, 2 - MULTIPAROUS	INTEGER
PREVIOUS C-SECTIONS	NUMBER	INTEGER
GA_WEEKS	GESTATIONAL AGE (WEEKS)	INTEGER
GA_DAYS	GESTATIONAL AGE (DAYS)	INTEGER
HEMORRHAGE RISK	1 - LOW RISK, 2 - MEDIUM RISK, 3 - HIGH RISK	INTEGER
ACTIVE BLEEDING ON ADMISSION	1- YES, 2 - NO	YES / NO
GESTATIONAL HYPERTENSION	1- YES, 2 - NO	YES / NO
CHRONIC HYPERTENSION	1- YES, 2 - NO	YES / NO
PREECLAMPSIA	O - NO 1- MILD, 2 - SEVERE	INTEGER
ECLAMPSIA	1- YES, 2 - NO	YES/ NO
THROMBOCYTOPENIA	1- YES, 2 - NO	YES / NO
USE OF ANTICOAGULANTS	1- YES, 2 - NO	YES / NO
PLACENTA_ACCRETA	1- YES, 2 - NO	YES / NO
PLACENTA PREVIA	1-YES, 2-NO	YES/ NO
DIABETES	1- YES, 2 - NO	YES / NO
MED_OXYTOCIN_1ST	1- YES, 2 - NO	YES / NO
MED_OXYTOCIN_2ST	1- YES, 2 - NO	YES / NO
MODE OF DELIVERY	1 - VAGINAL BIRTH, 2 - C-SECTION	YES / NO
RETAINED PLACENTA	1- YES, 2 - NO	YES / NO
PLACENTAL ABRUPTION	1- YES, 2 - NO	YES / NO
PERINEAL LACERATIONS	0 - NONE, 1 - 1° DEGREE, 2 - 2° DEGREE, 3 - 3° DEGREE, 4 - 4° DEGREE	INTEGER
FORCEPS	0 - NO, 1 - YES	YES / NO
ANALGESIA	1- YES, 2 - NO	YES / NO
NEWBORN_BIRTHWEIGHT	BIRTH WEIGHT (G)	300 - 6000
